# Portal vein recanalization and embolization of the transsplenic puncture tract using an Amplatzer® vascular plug: a case report

**DOI:** 10.1186/s13104-015-1138-4

**Published:** 2015-05-08

**Authors:** Ulrich Grosse, Klaus Brechtel, Dominik Ketelsen, Roland Syha, Gerd Grözinger, Fabian Springer, Christoph Thomas

**Affiliations:** Department of Diagnostic and Interventional Radiology, University Hospital Tuebingen, Hoppe-Seyler-Strasse 3, 72076 Berlin, Tuebingen Germany

**Keywords:** Transsplenic approach, Angioplasty, Portal vein occlusion

## Abstract

**Background:**

A transsplenic access for the catheterization of the portal venous system to treat a portal vein thrombosis and/or stenosis entails the risk of intra-abdominal or intrasplenic bleeding complications and has to be seen as an approach of last resort. This is one of few reported cases in the literature where a transsplenic puncture tract was successfully embolized using an Amplatzer® vascular plug 4 (8 mm; St. Jude Medical).

**Case presentation:**

This is the case report of a 58 years old Caucasian male patient who had received right sided extended hemihepatectomy with partial resection of the portal vein due to hilar cholangiocarcinoma three years ago. The patient suffered from portal hypertension with difficult controllable bleeding of esophageal varices due to chronically progressive thrombosis of the portal vein caused by chronic anastomosis stenosis of the reconstructed left portal vein branch (confirmed in a Magnetic Resonance Imaging (MRI) examination 6 months after the portal vein reconstruction). A transsplenic access (6 French) was chosen to allow recanalization of the portal vein, stent-angioplasty of the anastomosis and coiling of the gastric varices. The transsplenic tract was successfully embolized with an Amplatzer® Vascular Plug 4 and gelfoam pledgets.

**Conclusion:**

Amplatzer® Vascular plugs in combination with gelatin sponges can be used to efficiently and precisely seal transsplenic puncture sites.

**Electronic supplementary material:**

The online version of this article (doi:10.1186/s13104-015-1138-4) contains supplementary material, which is available to authorized users.

## Background

Several approaches are available for the catheterization of the portal venous system: the percutaneous transhepatic approach, the direct catheterization of a mesenteric vein via mini laparotomy, the use of a transjugular intrahepatic stent shunt (TIPSS) as well as access over a recanalization of the umbilical vein. Besides these options, the transsplenic approach provides a straightforward way (in the absence of a tortuous splenic vein) to access the portal venous system as well as gastric or oesophageal varices [1]. Nevertheless, a transsplenic access route has to be seen as an approach of last resort and complications in the form of intra-abdominal or intrasplenic bleeding might require an open surgical conversion.

We report a case of a successful recanalization of a chronic portal vein thrombosis via a transsplenic access route in a patient with stenosis of the reanastomized portal vein after extended right hemihepatectomy and reconstructed left portal vein. Similar to a recently published case by *Dollinger et al.*, an Amplatzer® plug 4 (8 mm; St. Jude Medical) in combination with gelfoam pledgets was successfully used to seal the transsplenic access site [2].

## Case presentation

A 58 years old Caucasian patient with a past medical history of a hilar cholangiocarcinoma (Bismuth stage IV) had received a right sided extended hemihepatectomy (segments 1, 4–8) with a reconstruction of the left proximal portal vein using a short, approximately 4 cm long allogenic iliac artery interposition graft three years earlier. Up to now no cancer recurrence was noted. However, due to a stenosis of the portal vein anastomosis which was suspected in early postoperative ultrasound examinations and diagnosed in a MRI examination 6 months after the operation (as demonstrated in Additional file 1: Figure S1) a chronically progressive thrombosis of the portal vein and consecutive portal hypertension had developed. At the time of the intervention, the patient suffered from recurrent bleeding of esophageal varices. No operative treatment options such as the creation of a porto-venous shunt or the revision of the anastomosis were feasible due to the postoperative situs.

**Figure 1:**
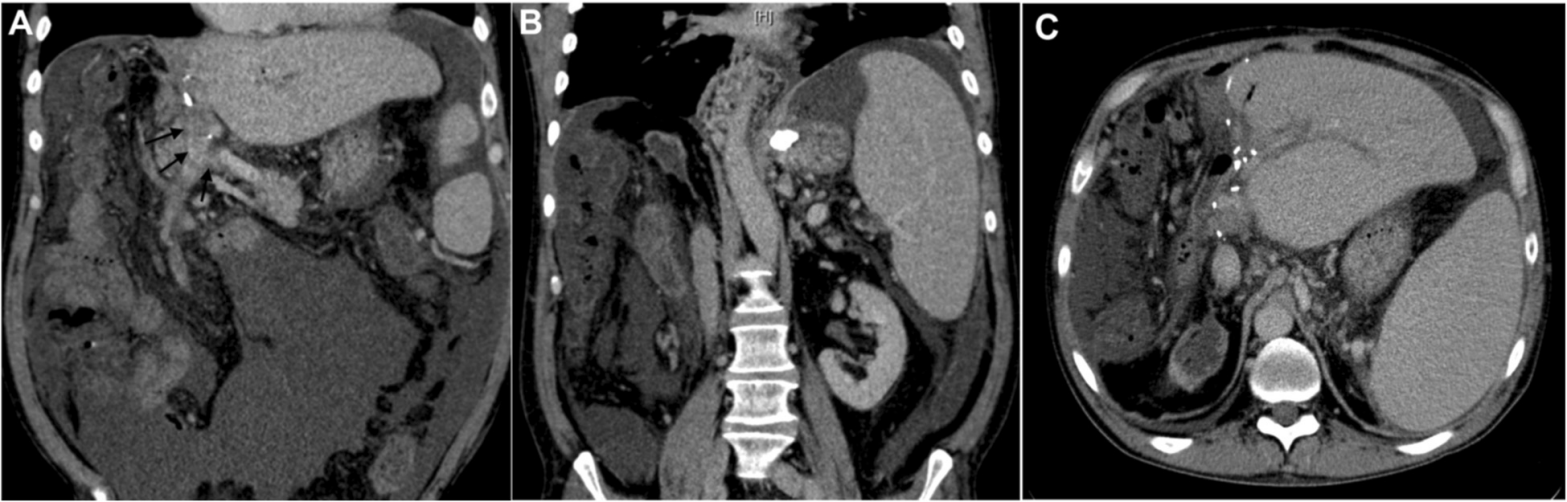
Pre-interventional Contrast-enhanced CT of the abdomen: **A**, **B**, **C** Coronal (arrows are pointing to the occluded portal vein) and axial venous phase images demonstrate the postoperative situs after hemihepatectomy, the cavernous portal vein occlusion with extension to the confluence, ascites and splenomegaly.

A pre-interventional contrast-enhanced CT of the abdomen (Figure 1A-C) demonstrates the situs after right sided hemihepatectomy, ascites, and the cavernous portal vein occlusion with extension of the thrombosis to the portal vein confluence as well as huge esophagogastric varices mainly arising from the left gastric vein. Laboratory data showed thrombocytopenia (54.000/μl), a slightly elevated INR value of 1.4 and a decreased hemoglobin level of 9.7 g/dL. ALT (12 U/l), NH3 (36 mg/dl) and total bilirubin (1.1 mg/dl) were unremarkable prior to the intervention.

**Figure 2:**
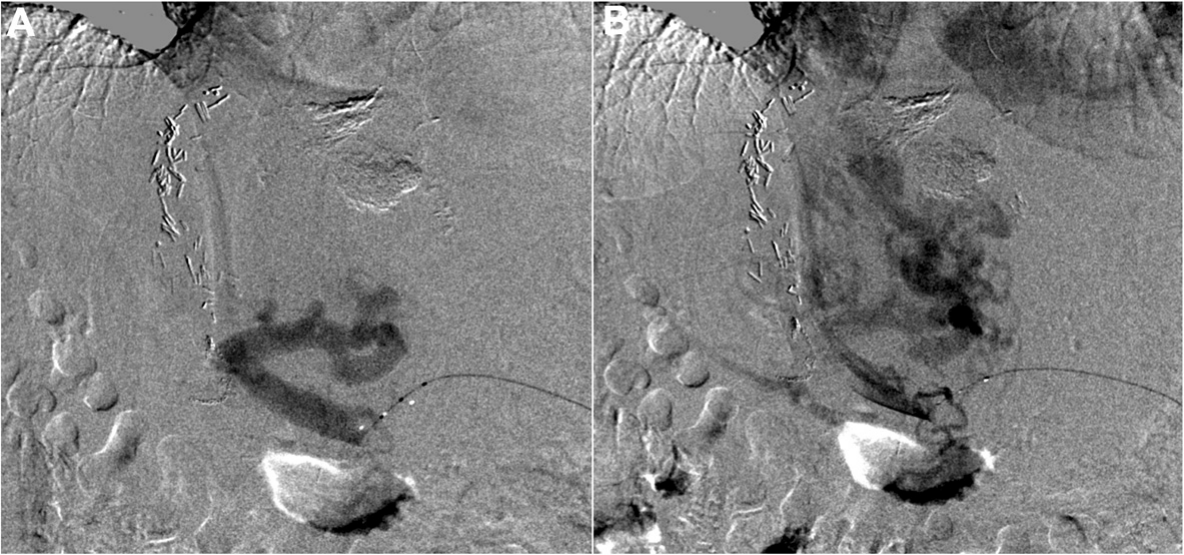
Baseline splenoportography: **A**, **B** Initial splenoportogram with an advanced support catheter (TrailBlazer™ 0.018 inch; ev3 Endovascular) confirmed a complete portal vein occlusion and showed massive esophageal and gastric varices.

**Figure 3:**
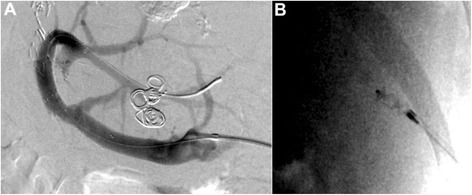
Splenoportography after stent extension and transsplenic tract embolization at the end of the intervention: **A** Final splenoportogram demonstrated a fast runoff of the applied contrast media in the intrahepatic portal vein branches and a reduced blood flow to the gastric varices after Coil-embolization. **B** Transsplenic tract embolization with an Amplatzer® plug 4 (8 mm; St. Jude Medical) as well as gelfoam pledgets delivered via the sheath as it was removed.

Due to the right-sided extended hemihepatectomy and the narrow poststenotic left portal venous branches catheterization of the portal venous system via a transjugular intrahepatic stent shunt (TIPSS) or a percutaneous transhepatic approach were considered inappropriate and a transsplenic approach was chosen. Under local anesthesia, a sonographically guided puncture (21 G fine needle) of a caudal hilar splenic vein was performed. Using Seldinger technique, a 0.018“ wire (V18 Control wire; Boston Scientific) and support catheter (TrailBlazer™, a 4 F catheter tapered down to 0.018 inch; ev3 Endovascular) were introduced. The following splenoportography (Figure 2A, B; note the faint contrast of the initial splenoportogram due to small end hole of the TrailBlazer™ catheter) confirmed a complete portal vein occlusion and showed massive esophageal and gastric varices. The portal vein occlusion could not be traversed using the 0.018” wire and the support catheter, thus a 4 F sheath and an JR4 configured 4 F angiography catheter (Cordis Corporation) with an hydrophilic guide wire (Terumo® 0.035”, Terumo Corp.) were advanced to improve controllability and pushability. After successful crossing of the portal vein occlusion, direct pressure measurements in the splenic vein showed a pressure of 32 mmHg, while 15 mmHg could be measured in the intrahepatic portal vein branches. After the initial angioplasty of the portal vein anastomosis and preceding segments (the main portal vein below the graft) with a 7 mm angioplasty balloon (Passeo-18; Biotronic) marked recoiling was observed. After upgrading the 4 F sheath to 6 F, the main portal vein was dilated with a 10 mm angioplasty balloon (Armada™ 35; Abbott Vascular). Due to a still evident recoiling, stent-angioplasty of the anastomosis with a 10/60 mm nitinol stent (Protégé GPS™; ev3 Endovascular) and post dilatation with a 10 mm balloon were performed. The following splenoportography showed mobile parts of the thrombus proximal to the implanted stent making a dual stent extension with a 10/60 mm (Protégé GPS™; ev3 Endovascular) and a 12/40 mm nitinol stent (Sinus-SuperFlex™; OptiMed) to preceding portal vein segments necessary. The finally acquired splenoportography after coiling of the gastric varices demonstrated a satisfying result with a fast runoff of the applied contrast media in the intrahepatic portal vein branches (Figure 3A). Obtained pressure values after stent angioplasty showed 32 mmHg in the splenic vein and 22 mmHg in intrahepatic portal vein branches, but due to the fast runoff of the applied contrast media no further angioplasty was carried out.

At the end of the intervention, the transsplenic tract was embolized with an Amplatzer® plug 4 (8 mm; St. Jude Medical) (Figure 3B) as well as gelfoam pledgets. The technique used in this case was similarly to the one described by *Dollinger et al.* in a recent published case series [2]: While the Amplatzer® plug was fixed in the desired position approximately 2 cm medial to the splenic capsule, the 6 F sheath was slowly pulled back. Contrast medium was delivered over the sheath to verify the correct position of the Amplatzer® plug before it was released. Due to the poor condition of the patient and the increased pressure values in the splenic vein, gelfoam pledgets were additionally delivered via the sheath as it was removed.

**Figure 4:**
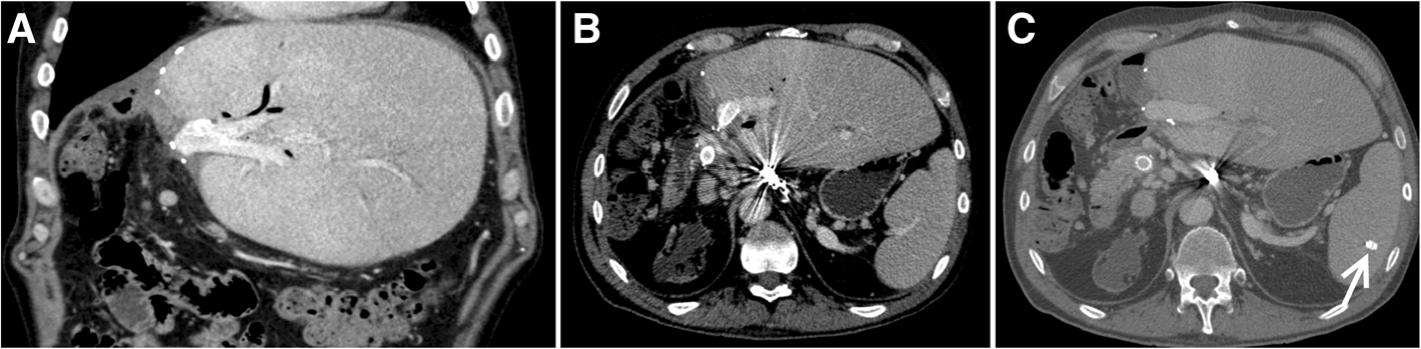
Contrast-enhanced PET-CT of the abdomen 3 years post-interventional: **A**, **B**, **C** Coronal and axial venous phase images demonstrate a patent portal vein with stents in situ, a left hepatic lobe hypertrophy and the Amplatzer® vascular plug (marked with an arrow in C) within the spleen.

A post-interventional ultrasound examination showed no evidence of a hemoperitoneum or a perisplenic or splenic hematoma formation. Nevertheless, as an expected minor complication leakage of a larger quantity of ascites at the puncture site occurred in the first hours after the intervention. ALT (7 U/l), NH3 (30 mg/dl) and total bilirubin (0.6 mg/dl) remained unremarkable the following days and no bleeding of the esophageal varices reoccurred after the intervention. An esophageal gastroduodenoscopy was performed 5 months later and could not detect esophageal varices. Regular follow-up ultrasound examinations were carried out on an outpatient basis and demonstrated a patent portal vein Three years after the recanalization of the portal vein a PET-CT was performed due to recurrent cholangitis (Figure 4), which showed no malignant recurrence, demonstrated a patent portal vein with left hepatic lobe hypertrophy (Figure 4) and the Amplatzer® vascular plug within the spleen (Figure 4C).

## Conclusion

In this patient, a thrombotic stenosis of the reconstructed left portal vein anastomosis (diagnosed in a MRI examination 6 months after the operation (as demonstrated in Additional file 1: Figure S1) after right-sided extended hemihepatectomy was the triggering reason for the portal hypertension. In general, the exact cause of a portal vein thrombosis often remains unclear although risk factors such as malignancy, thrombophilic syndromes, myeloproliferative disorders, pregnancy, local inflammation and infection are known [3]. Even though less than 3% of all cases of portal hypertension are caused by portal vein thrombosis, a population-based study by *Ögren et al.* has suggested that the prevalence of portal vein thrombosis might be underestimated as the authors found an occurrence of up to 14% in patients with cirrhosis and hepatic cancer as well as a lifetime risk of 1% in the general population [4].

In the context of postoperative complications, *Yoshiya et al.* presented the first larger study on postoperative portal vein thrombosis after hepatectomy and concluded that this complication is not rare and occurred in 9.1% of all their investigated cases [5]. Furthermore, the authors of this article concluded that besides of above mentioned risk factors the duration of clamping the hepatoduodenal ligament (“Pringle maneuver”) represented another significant risk factor [5].

Clinically, an acute (non-cavernous) form with abdominal pain, fever, nausea, ascites and variceal bleeding can be distinguished from a chronic (cavernous) form with symptoms of portal hypertension lead to such as ascites, portosystemic collateralization with formation of varices and/ or hepatic encephalopathy [6]. The therapeutic management of an acute portal vein thrombosis is based on the clinical presentation of the patient and includes systemic anticoagulation as well as local thrombolysis, mechanical thrombectomy, angioplasty, a combination of these or the creation of a transjugular intrahepatic portosystemic stent shunt (TIPSS) in more symptomatic patients (for more detailed information please refer to the review by *Lang et al.*) [7].

On the contrary, there is less evidence in the literature regarding the best therapeutic approach in symptomatic patients with a cavernous transformation of the portal vein although several studies have successfully demonstrated the feasibility of a endovascular portal vein recanalization using different access routes [1,6,8].

Especially in the case of portal hypertension, transsplenic access for the catheterization of the portal venous system entails the risk of intra-abdominal or intrasplenic bleeding complications and has to be seen as an approach of last resort. *Gong et al.* reported that in a series of 18 patients, transsplenic access using a 5 F sheath led to a hemoperitoneum in two patients while *Liang et al.* experienced three cases of significant bleeding in a series of 17 patients [9,10]. Nevertheless, *Probst et al.* first reported a simple technique to avoid these bleeding complications from the puncture site by plugging the splenic access tract with compressed gelatin sponges in 1978 [11]. Nowadays different embolization techniques have been developed and subsequently reduced the risk of splenic hemorrhage [12]. For example *Chu et al.* used 4 to 9 F sheaths to access the splenic vein and reported no bleeding complications in nine patients by using different coils in combination with a mixture of n-butyl cyanoacrylat (Histoacryl) and lipiodol for the embolization of the splenic puncture site [1]. Using a similar embolization technique *Zhu et al.* reported in a larger clinical study on 46 patients, 3 cases with major and 7 cases with minor bleeding complications after a percutaneous transsplenic approach with 5 F sheaths [8]. In a recent published case series (5 patients) *Dollinger et al.* used Amplatzer® vascular plugs 2 and 4 (4 – 8 mm) to embolize large bore (6 – 10 F sheaths) and/or short transparenchymal hepatic (4 cases) or splenic (1 case) puncture tracts [2]. Although, the only patient with a transsplenic access in this study died 21 days after the intervention due to pneumonia, the author did not experience bleeding complications from the hepatic and splenic puncture tracts. As a minor complication one patient developed a postinterventional focal liver abscess adjacent to the Amplatzer® plug, so that the authors recommended a periinterventional antimicrobial prophylaxis.

In summary, low-profile sheaths or catheters make the occurrence of life-threatening bleeding complications after a transsplenic or transhepatic access highly improbable. In the presented case a 6 F sheath had to be used due to the required stent implantation in the portal vein. Consecutively, the splenic access tract was embolized using an Amplatzer® Vascular Plug 4 (8 mm; St. Jude Medical) in an off-label scenario in combination with gelatin sponges, which allowed efficient and precise sealing of the splenic puncture site.

## Consent

Written informed consent was obtained from the patient for publication of this case report and accompanying images. A copy of the written consent is available for review by the Editor-in-Chief of this journal.

## Additional file

Additional file 1: Figure S1. Contrast-enhanced MRI using a T1-w 3D fast low angle shot (FLASH) fat supressed sequence 6 months postoperative: **A, B** Consecutive axial portal venous phase MRI images demonstrating a stenosis of the reconstructed left portal vein anastomosis (arrows are pointing to the anastomotic stenosis). Note that susceptibility artefacts are caused by postoperative clips at the anastomosis site.

## Abbreviations

ALT: alanine aminotransferas
FLASH: fast low angle shot
INR: International normalized ratio
MRI: Magnetic Resonance Imaging
NH3: Ammonia
PET-CT: positron emission tomographic (PET) computed tomography (CT)
TIPSS: Transjugular intrahepatic portosystemic stent shunt
