# Supplementary figures and images for: Portal vein recanalization and embolization of the transsplenic puncture tract using an Amplatzer® vascular plug: a case report
Source: BMC Res Notes. 2015 May 8;8:193. doi: 10.1186/s13104-015-1138-4 (PMC4429671; doi:10.1186/s13104-015-1138-4)

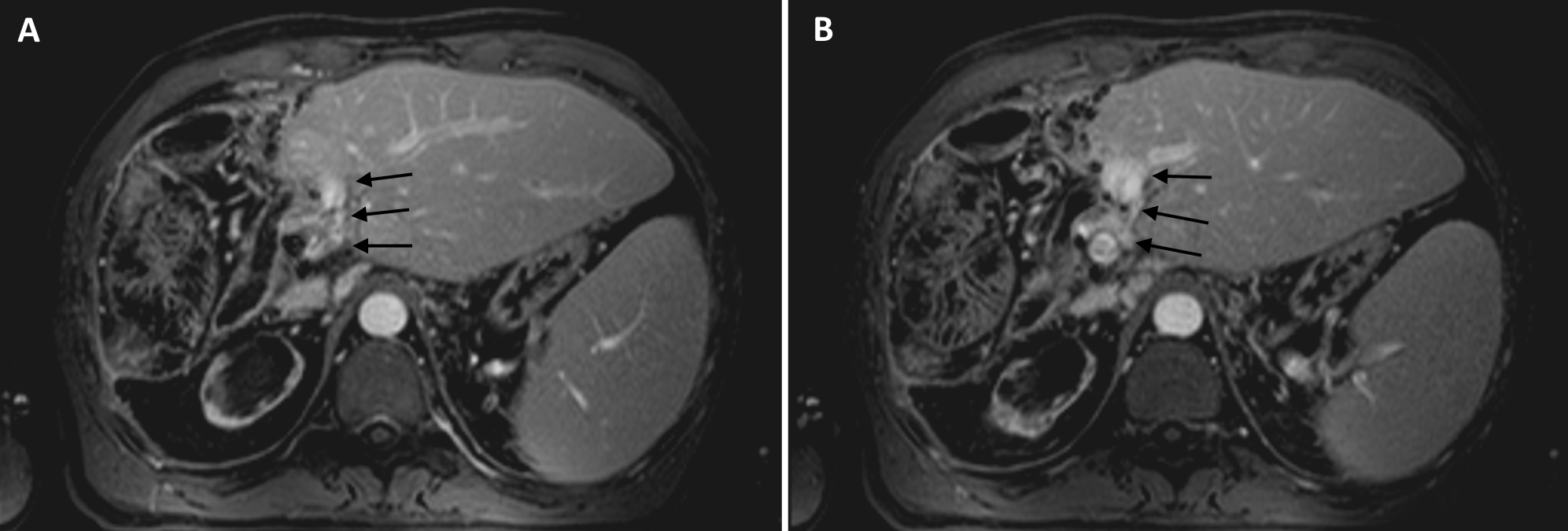

Supplement: Additional file 1: Figure S1. — Contrast-enhanced MRI using a T1-w 3D fast low angle shot (FLASH) fat supressed sequence 6 months postoperative: A, B Consecutive axial portal venous phase MRI images demonstrating a stenosis of the reconstructed left portal vein anastomosis (arrows are pointing to the anastomotic stenosis). Note that susceptibility artefacts are caused by postoperative clips at the anastomosis site. [file 13104_2015_1138_MOESM1_ESM.tiff]
